# Anti-tumor activity of anthrax toxin variants that form a functional translocation pore by intermolecular complementation

**DOI:** 10.18632/oncotarget.17729

**Published:** 2017-05-09

**Authors:** Shihui Liu, Qian Ma, Rasem Fattah, Thomas H. Bugge, Stephen H. Leppla

**Affiliations:** ^1^ Proteases and Tissue Remodeling Section, National Institute of Dental and Craniofacial Research, National Institutes of Health, Bethesda, MD 20892, USA; ^2^ Microbial Pathogenesis Section, Laboratory of Parasitic Diseases, National Institute of Allergy and Infectious Diseases, National Institutes of Health, Bethesda, MD 20892, USA

**Keywords:** anthrax protective antigen, lethal toxin, MMPs, protein delivery, tumor targeting

## Abstract

Anthrax lethal toxin is a typical A-B type protein toxin secreted by *Bacillus anthracis*. Lethal factor (LF) is the catalytic A-subunit, a metalloprotease having MEKs as targets. LF relies on the cell-binding B-subunit, protective antigen (PA), to gain entry into the cytosol of target cells. PA binds to cell surface toxin receptors and is activated by furin protease to form an LF-binding-competent oligomer-PA pre-pore, which converts to a functional protein-conductive pore in the acidic endocytic vesicles, allowing translocation of LF into the cytosol. During PA pre-pore-to-pore conversion, the intermolecular salt bridge interactions between Lys397 and Asp426 on adjacent PA protomers play a critical role in positioning neighboring luminal Phe427 residues to form the Phe-clamp, an essential element of the PA functional pore. This essential intermolecular interaction affords the opportunity to create pairs of PA variants that depend on intermolecular complementation to form a functional pore. We have previously generated PA variants with furin-cleavage site replaced by substrate sequences of tumor-associated proteases, such as urokinase or MMPs. Here we show that PA-U2-K397Q, a urokinase-activated PA variant with Lys397 residue replaced by glutamine, and PA-L1-D426K, a MMP-activated PA variant with Asp426 changed to lysine, do not form functional pores both *in vitro* or *in vivo* unless they are used together. Further, the mixture of PA-U2-K397Q and PA-L1-D426K displayed potent anti-tumor activity in the presence of LF. Thus, PA-U2-K397Q and PA-L1-D426K form a novel intermolecular complementation system with toxin activation relying on the presence of two distinct tumor-associated proteases, i.e., urokinase and MMPs.

## INTRODUCTION

Anthrax lethal toxin has become one of the best-characterized protein delivery systems, one that can be modified to selectively target cancer [1–8]. The toxin is an A-B type toxin, with protective antigen (PA, the B subunit) as the cell binding moiety and lethal factor (LF, the A subunit) as the effector component. To intoxicate a target cell, PA binds to its specific cell surface receptors, tumor endothelium marker-8 (TEM8) and/or capillary morphogenesis protein-2 (CMG2) [9, 10]. An aminoterminal fragment (PA20) from receptor-bound PA is then proteolytically removed by ubiquitous furin or furin-like proteases, resulting in formation of an LF-binding-competent PA oligomer (heptamer or octamer), also called PA pre-pore [11]. The complex of PA pre-pore and LF enters cells by receptor-mediated endocytosis. Within endosomes, the acidic environment causes conformational changes of the PA oligomer, resulting in membrane insertion and conversion of the PA pre-pore to a functional pore [12–14], which actively translocates LF into the cytosol to exert its cytotoxic effects [11, 15–17]. LF is a zinc-metalloproteinase that inactivates mitogen-activated protein kinase kinases (MEKs) [18, 19], thereby shutting down the RAS-RAF-MEK-ERK pathway, which is essential for tumor angiogenesis [1] and is frequently over-activated in human cancers, due to oncogenic mutations in the components in this pathway such as the B-RAF V600E mutation present in about 60% of human melanomas [20].

The PA pore plays an active transporter role in the translocation of LF. During PA pore formation, seven (eight for octamer) luminal Phe427 residues in domain 2 converge to form a narrow and hydrophobic constriction, the so-called Phe-clamp, which is believed to create a hydrophobic environment that transiently interacts with the molten globular hydrophobic segments of the unfolded LF, thereby reducing the energy penalty of exposing the hydrophobic side chains of LF to the solvent [13, 14]. Through interacting with unfolded, passing LF, the narrow Phe-clamp also forms a perfect seal around the translocating polypeptide, preventing the passage of ions, and thereby maintaining the proton gradient, a driving force for LF translocation across the endosomal membrane [14].

During PA pre-pore-to-pore conversion, the salt bridge interactions formed between Lys397 and Asp426 on adjacent PA protomers play an essential role in positioning Phe427 to form the Phe-clamp [12]. This essential intermolecular interaction affords the opportunity to make functional PA pore formation dependent on intermolecular complementation of two PA variants that each depend on distinct tumor-associated proteases for activation. We have previously generated PA variants with furin cleavage site replaced by substrate sequences of tumor-associated proteases, such as urokinase or MMPs [21, 22]. Here we show that PA-U2-K397Q, a urokinase activated PA variant with Lys397 residue replaced by glutamine, and PA-L1-D426K, a matrix metalloproteinase (MMP)-activated PA variant with Asp426 changed to lysine, could not form functional pore when used alone, but were able to complement to form an active PA oligomer when used together both *in vitro* and *in vivo*. Further, the mixture of PA-U2-K397Q and PA-L1-D426K displayed potent anti-tumor activity in the presence of LF. Thus, PA-U2-K397Q and PA-L1-D426K form a novel intermolecular complementation system with activation relying on the presence of two distinct tumor-associated proteases, i.e., urokinase and MMPs.

## RESULTS

### PA variants defective in functional pore formation

**Figure 1:**
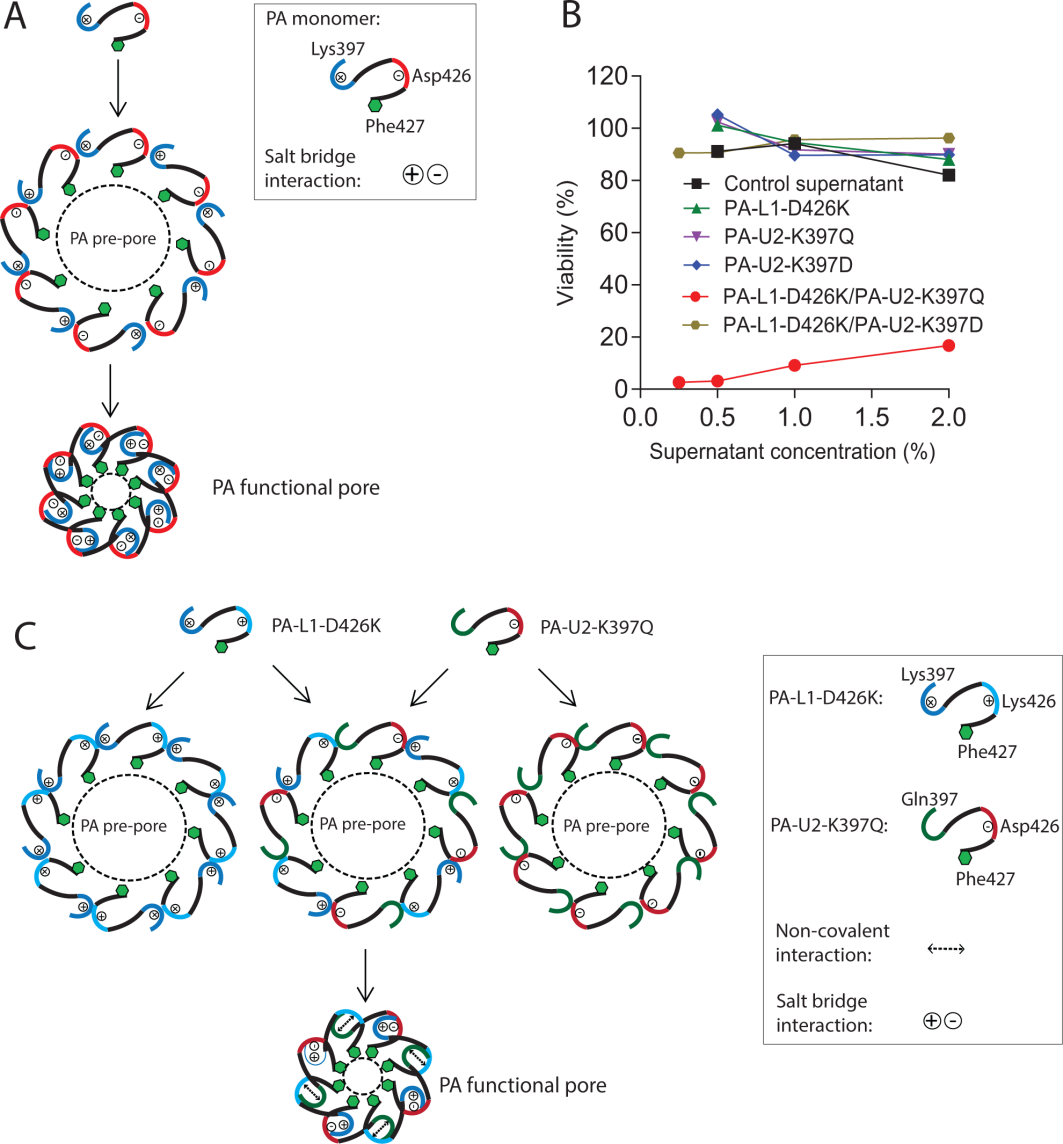
PA variants with defects in functional pore formation **(A)** Schematic representation of the role of the interaction between residues Lys397 and Asp426 from adjacent PA protomers in PA functional pore formation. For simplicity, only octamers are shown. **(B)** Cytotoxicity of concentrated culture supernatants from PA variant-expressing BH480 strains. B16-BL6 cells cultured in 96-well plates were treated with various dilutions of the concentrated supernatants, or the indicated combinations of the supernatants from the PA variant-transformed BH480 strains, in the presence of 100 ng/ml FP59 for 48 h. MTT assay was used to evaluate cell viabilities relative to the non-toxin treated cells. Concentrated supernatant from a non-transformed BH480 strain was used as a control. Only the combination of PA-L1-D426K and PA-U2-K397Q demonstrated toxicity to the cells in the presence of FP59. **(C)** Schematic representation of the intermolecular complementation abilities of PA-L1-D426K and PA-U2-K397Q in PA functional pore formation. No functional pore is formed when PA-L1-D426K or PA-U2-K397Q is used alone. The functional pore shown is an idealized one in which the two PA variants alternate in the structure, whereas in reality they are expected to be randomly arranged.

The salt bridge interaction between the negatively-charged residue Asp426 and the positively-charged residue Lys397 from adjacent PA protomers is crucial to form a functional protein-conductive pore [12], allowing translocation of the loaded effector proteins into the cytosol (Figure 1A). To explore this fact to design PA variants requiring intermolecular complementation to form functional pores, we first used a charge reversal strategy by mutating Lys397 to Asp in the urokinase-activated PA variant (PA-U2) and Asp426 to Lys in the MMP-activated PA variant (PA-L1) [21, 22]. The resulting PA variants PA-U2-K397D and PA-L1-D426K were expressed in an avirulent *B. anthracis* strain BH480 [23, 24]. Although the U2 and L1 sequences add 3 and 4 residues, respectively, at the protease cleavage site, we identify residues by their locations in the native sequence (e.g., K397) rather than in the actual PA-U2 and PA-L1 proteins (where they are K400 and K401, respectively). In the BH480 expression system, PA proteins are efficiently secreted as the major protein component of the culture medium, allowing quick evaluation of their activity without further purification. The activities of PA proteins were tested by combining them with FP59 - a fusion protein of LF amino acids 1–254 and the catalytic domain of *Pseudomonas aeruginosa* exotoxin A that kills cells by ADP-ribosylation of eEF2 after delivery to the cytosol [25]. As expected, PA-U2-K397D and PA-L1-D426K, when used individually with FP59, could not kill B16-BL6 melanoma cells (Figure 1B), which express both urokinase and MMPs [1]. Furthermore, when PA-U2-K397D and PA-L1-D426K were administered together with FP59, B16-BL6 cells remained refractory to killing, indicating that the two PA mutants could not complement to form a functional pore able to translocate FP59. This suggested that although Asp397 in PA-U2-K397D and Lys426 in PA-L1-D426K could potentially form a salt bridge interaction, either one or both mutations may compromise the structural integrity of PA.

Because the homologues of PA from *Clostridium* species have Gln at the corresponding position of Lys397 in PA and because a PA variant simultaneously containing K397Q and D426K substitutions is functional [12], we generated the PA variant PA-U2-K397Q, which is urokinase-activated, and examined its ability to complement the MMP-activated PA-L1-D426K to form an active PA oligomer (Figure 1B). Just like PA-L1-D426K and PA-U2-K397D, PA-U2-K397Q was not toxic to B16-BL6 cells in the presence of FP59. However, PA-U2-K397Q and PA-L1-D426K were toxic to the cells when administered together in the presence of FP59 (Figure 1B and 1C). Thus, PA-U2-K397Q and PA-L1-D426K constitutes a novel intermolecular complementation system with activation relying on the presence of two different kinds of tumor-associated proteases, i.e., urokinase and MMPs.

### Intermolecular complementing activity of PA-U2-K397Q and PA-L1-D426K

**Figure 2:**
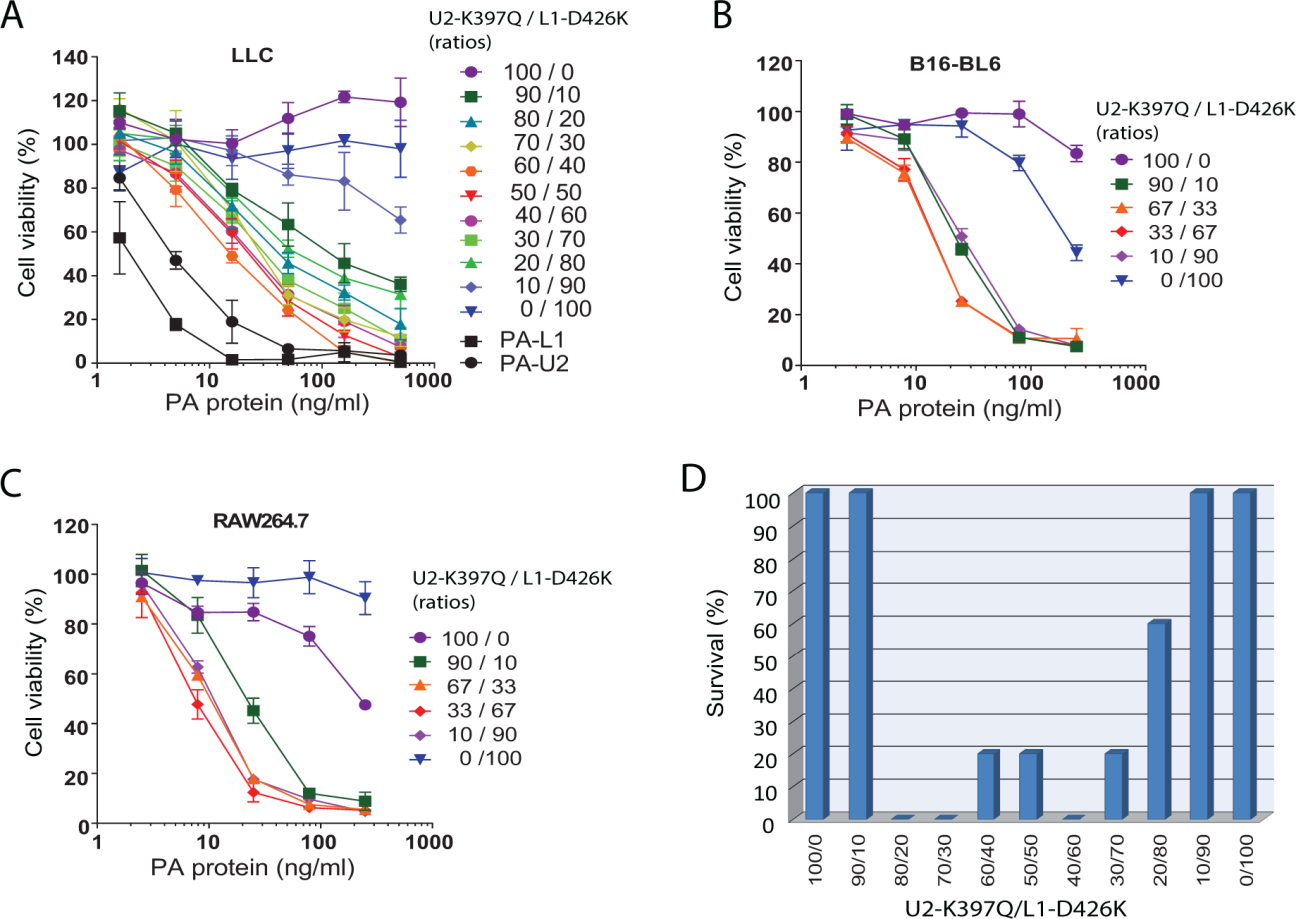
Intermolecular complementation activities of PA-U2-K397Q and PA-L1-D426K **(A-C)** Intermolecular complementation cytotoxicity of PA-U2-K397Q and PA-L1-D426K. LLC cells (A) and B16-BL6 melanoma cells (B) were incubated with various concentrations of the indicated ratios of purified PA-U2-K397Q and PA-L1-D426K in the presence of FP59 (100 ng/ml) for 48 h, followed by MTT assays to determine cell viabilities. Parental PA variants PA-L1 and PA-U2 were included in (A) for comparison. RAW267.4 cells (C) were incubated with various concentrations of the indicated ratios of PA-U2-K397Q and PA-L1-D426K in the presence of LF (500 ng/ml) for 5 h, followed by MTT assays to determine cell viabilities. **(D)** *In vivo* intermolecular complementation toxicity of PA-U2-K397Q and PA-L1-D426K. Mice (tumor-free) were challenged with two intraperitoneal doses of the indicated ratios of 20 μg total of PA-U2-K397Q and PA-L1-D426K plus 10 μg FP59 with a two-day interval. Survival was monitored at regular intervals and the survival after two weeks is shown.

To further characterize PA-U2-K397Q and PA-L1-D426K, these PA variants were purified to homogeneity. Various concentrations and ratios of PA-U2-K397Q and PA-L1-D426K were added to B16-BL6 melanoma cells as well as to mouse Lewis lung carcinoma cells (LLC) in the presence of FP59 (Figure 2A and 2B). Whereas PA-U2-K397Q and PA-L1-D426K showed no or low toxicity to LLC and B16-BL6 cells when used alone, the combination of the two PA variants displayed cytotoxicity to both tumor cell lines, with the highest activity observed with ratios close to 1:1. However, the cytotoxicity of the combination was about 10-fold lower than their parental variants PA-U2 and PA-L1. This may be explained by the fact that a fraction of the heterogeneous population of PA oligomers contains unproductive intersubunit contacts from adjacent identical protomers.

To examine the intermolecular complementing activity of PA-U2-K397Q and PA-L1-D426K *in vivo*, we challenged mice (tumor-free) with two systemic doses of various ratios of a total of 20 μg of PA-U2-K397Q and PA-L1-D426K plus 10 μg FP59. When used alone or in the extreme ratios of 9:1 and 1:9, PA-U2-K397Q and PA-L1-D426K did not cause mortality to mice (Figure 2D). However, when these PA variants were used in ratios from 80:20 to 20:80, mortalities were observed. While 100% mortality was observed with the ratio of 80:20 of PA-U2-K397Q/PA-L1-D426K, only 40% of mice succumbed to the ratio of 20:80 of PA-U2-K397Q/PA-L1-D426K, suggesting that the *in vivo* MMP activity is likely higher than urokinase activity in PA variant activation (Figure 2D).

### Anti-tumor activity of the intermolecular PA variants

**Figure 3:**
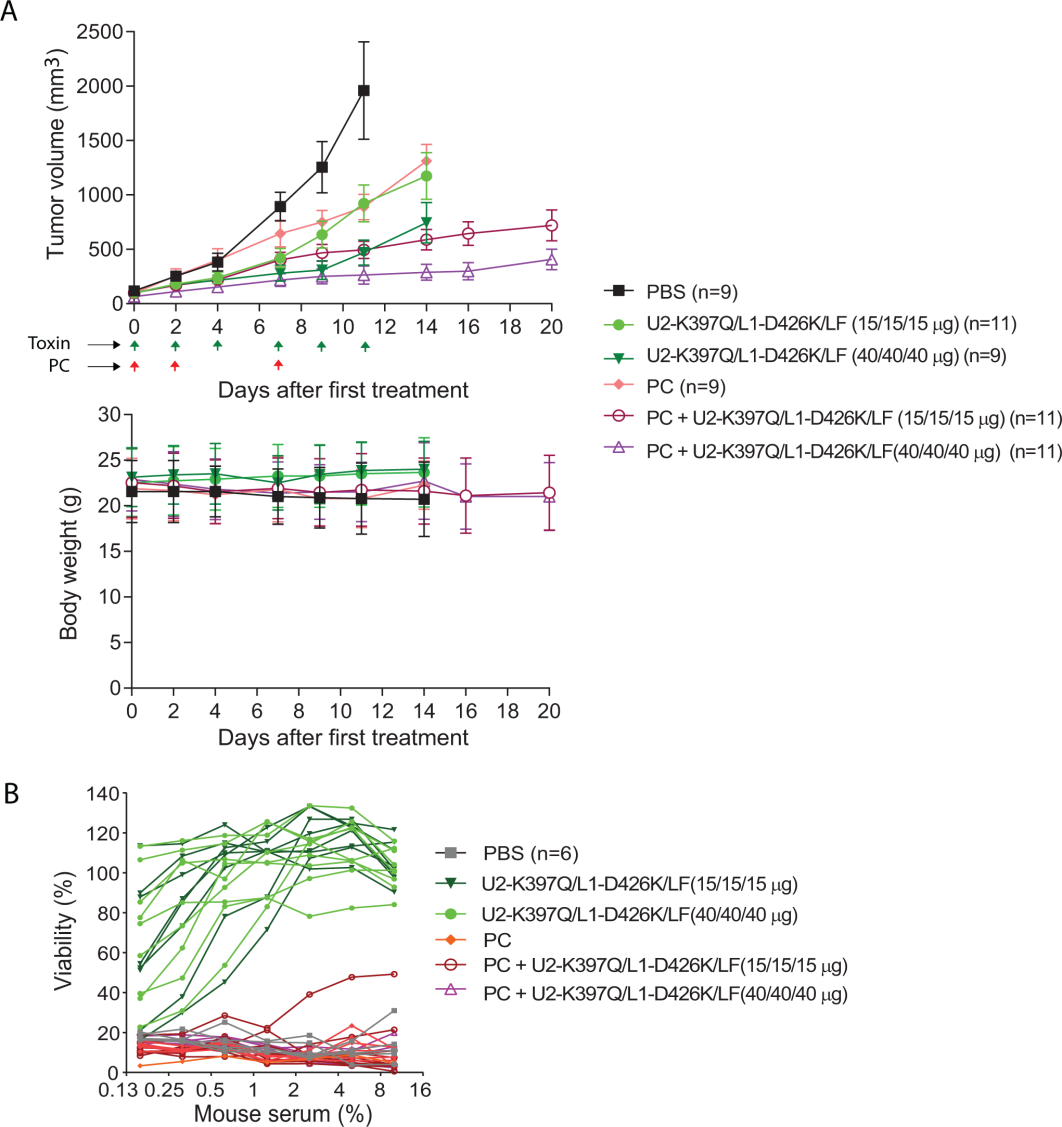
Anti-tumor activity of the engineered anthrax lethal toxin **(A)** LLC lung carcinoma-bearing immunocompetent C57BL/6J mice were treated intraperitoneally with PBS, 15 μg (low dose) of each of PA-U2-K397Q, PA-L1-D426K, and LF, or 40 μg (high dose) of each of PA-U2-K397Q, PA-L1-D426K, and LF. PC regimen (20 μg pentostatin and 1 mg cyclophosphamide) alone or in combination with low or high dose of the engineered toxins was also included. Schedules for PC and the toxin treatments are indicated by the arrows. Tumor volumes and body weights were monitored. Sera were collected from mice at the end of the experiments for analyses of the anti-toxin neutralizing antibody production in B. Tumor volumes, mean ± SE; Body weights, mean ± SD. One-way ANOVA analysis (two-tailed) for tumor size differences: PBS vs. all other groups, *P* < 0.05; PA-U2-K397Q/PA-L1-D426K/LF low dose vs. high dose, *P* = 0.03; PC+ PA-U2-K397Q/PA-L1-D426K/LF (low dose) vs. PA-U2-K397Q/PA-L1-D426K/LF (low dose), *P* = 0.02; PC+ PA-U2-K397Q/PA-L1-D426K/LF (high dose) vs. PA-U2-K397Q/PA-L1-D426K/LF (high dose), *P* = 0.05. **(B)** PC regimen efficiently prevents neutralizing antibody production against the intercomplementing toxins. RAW264.7 cells were incubated with PA/LF (100 ng/mL each) for 5 h in the presence of various dilutions of sera obtained from representative mice in (A). Cell viabilities were determined by the MTT assay as described in Methods. Note that no anti-toxin neutralizing antibodies were detected in the mice from the PC and PC + toxin combined therapy groups.

To evaluate the anti-tumor activity of the mixture of PA-U2-K397Q/PA-L1-D426K, a trial was performed using highly metastatic LLC (mouse) carcinomas established in syngeneic immunocompetent C57BL/6J mice. Because these engineered toxin proteins are foreign antigens to hosts, and because the combination of pentostatin and cyclophosphamide (PC), a regimen used to prevent host-versus-graft reactivity, is effective in preventing the production of anti-toxin neutralizing antibodies [1, 26, 27], we also included the PC regimen in the study. Therefore, the tumor-bearing mice were treated with either PBS, PC regimen, low (15 μg/15 μg/15 μg) or high (40 μg/40 μg/40 μg) dose of PA-U2-K397Q/PA-L1-D426K/LF, or the combined therapy of the PC regimen and low or high dose of PA-U2-K397Q/PA-L1-D426K/LF, following the schedule shown in Figure 3A. The engineered toxin alone showed a dose-dependent anti-tumor effect (Figure 3A). As shown previously [1], the PC regimen alone also exhibited anti-tumor activity (Figure 3A). Interestingly, the combined treatments showed higher anti-tumor efficacy than the single agent treatment groups (Figure 3A). As expected, neutralizing antibodies were detected in all the mice treated with toxin in the absence of PC at the end of the experiments (day 14). Strikingly, no neutralizing antibodies were detected in the tumor-bearing mice of the combined therapy groups (Figure 3B), explaining the higher anti-tumor efficacy observed in the combined treatments groups.

## DISCUSSION

The anthrax toxin protein delivery system is one of the best-characterized systems for delivery of polypeptides into cells. The system can be modified in multiple ways to achieve specific delivery of therapeutic effector proteins to certain cell types, including cancer cells [3–8]. In particular, we have generated PA variants that are dependent on tumor-associated proteases, such as urokinase or MMPs, for activation [21, 22, 28]. Upon proteolytic activation on the surface of a target cell, PA oligomerizes and gains the capacity to bind effector proteins such as LF. Each LF binding site is formed by subsites from two adjacent PA protomers [29–31]. Based on this feature, we have in the past successfully generated PA variants, i.e. PA-L1-I207R and PA-U2-R200A that depend on intermolecular complementation to form the active LF-binding sites [2, 4, 32]. Because these two PA variants require, respectively, MMPs and urokinase for activation, the action of this intermolecular complementation system relies on the presence of two distinct tumor-associated proteases, thereby achieving high tumor specificity.

In this work, we exploited another feature of the PA oligomer to design a novel intermolecular complementation system. PA pre-pore-to-pore conversion within the endocytic pathway is essential for effector protein translocation into the cytosol [13, 14]. During PA pre-pore-to-pore conversion, the salt bridge and hydrogen bond interactions between the oppositely charged residues Lys397 and Asp426 on adjacent PA protomers play an essential role in positioning the luminal Phe427 to form the Phe-clamp [12, 14]. We reasoned that PA variants with Lys397 or Asp426 replaced by an oppositely charged residue would be defective in PA functional pore formation, but randomly arranged heterogeneous oligomers containing both variants might form a functional pore through Asp397 and Lys426 interactions between adjacent different PA variants. However, when the two PA variants were combined they failed to form an active PA pore through their intermolecular complementation. One possibility is that one or both of the mutations may compromise the structural integrity of the PA oligomers. Because a Gln residue in several PA homologues from *Clostridium* species, such as the Sb component from *Clostridium spiroforme* and CdtB from *Clostridium difficile*, is present at the position corresponding to Lys397 in PA, and because a PA variant containing both K397Q and D426K substitutions is active [12], we examined whether PA-U2-K397Q could complement with PA-L1-D426K to form a functional pore. Indeed, although PA-U2-K397Q and PA-L1-D426K had little or no activity when used alone, they were able to kill tumor cells expressing both urokinase and MMPs when used in combination. Thus, PA-U2-K397Q and PA-L1-D426K form a novel intermolecular complementation system with activation relying on the presence of two distinct tumor-associated proteases, i.e., urokinase and MMPs.

As shown in Figure 1C, two different interactions would be expected to contribute to the Phe-clamp through PA-U2-K397Q and PA-L1-D426K intermolecular complementation: salt bridge interactions between Asp426 and Lys397 and a weaker non-covalent interaction (hydrogen bonds) between Lys426 and Gln397. However, the activity restored by PA-U2-K397Q and PA-L1-D426K intermolecular complementation did not reach the levels of their parental variants PA-U2 and PA-L1. There are at least two possibilities that may explain this: 1) the interaction between Lys426 and Gln397 is weaker than the native salt bridge interactions, and 2) a fraction of the heterogeneous population of PA oligomers may contain unproductive intersubunit contacts when adjacent protomers are of the same type. Although this novel intermolecular complementation system displays a lower activity compared to the parental PA variants PA-L1 and PA-U2, patients are also expected to be able to tolerate a correspondingly higher dose during treatment. Because the toxin receptors are expressed in normal endothelial cells and blood cells in circulation, the toxin may be sequestered on these cells before reaching tumor tissues (“the sink effect”). Engineered toxins that can be used at higher doses may have the ability to overcome this “sink effect”. In this regard, we did observe a potent dose-dependent anti-tumor activity of PA-U2-K397Q and PA-L1-D426K when combined with LF in LLC tumor-bearing mice.

Recently, a combined pentostatin and cyclophosphamide (PC) regimen, which is used clinically to treat chronic B-cell leukemia [33, 34], has been successfully used to prevent the induction of neutralizing antibodies against a *Pseudomonas aeruginosa* exotoxin A-based immunotoxin in human mesothelioma patients [26, 27]. We have also successfully used a PC regimen to prevent the production of antibodies against the engineered anthrax toxins in tumor-bearing mice [1]. Here, we further showed that this PC regimen could also effectively block antibody production against our novel intermolecular complementing PA variants with no need for pretreating the hosts prior to the toxin therapy. As expected, the combined therapy demonstrates synergistic effects in anti-tumor activity. The striking effects of the PC regimen in blocking induction of neutralizing antibodies against anthrax toxins appeared to exceed the effects on the exotoxin A-based immunotoxins [26, 27]. This may be due to the lack of pre-exposure of both humans and mice to *B. anthracis*, while a pre-exposure to the opportunistic pathogen *Pseudomonas aeruginosa* is common among both humans and experimental animals.

In summary, we have generated a mechanistically novel system of intercomplementing PA variants that provides potent and sustained anti-tumor activity when administered in combination with LF and a PC regimen.

## MATERIALS AND METHODS

### Construction and purification of PA variants

PA-L1 is an MMP-activated PA variant, in which the furin-cleavage sequence RKKR (residues 164-167) is replaced with a MMP substrate sequence GPLGMLSQ [22]. PA-U2 is a urokinase-activated PA variant with furin site replaced with an artificial urokinase substrate sequence PGSGRSA [21, 28]. The Q5 site-directed mutagenesis kit (Cat. No. E0554S, New England Biolabs, Ipswich, MA) was used to generate PA-U2-K397D and PA-U2-K397Q using PA-U2-expressing plasmid pYS-PA-U2 as a template, and PA-L1-D426K using PA-L1-expressing plasmid pYS-PA-L1 as a template. The resulting PA variant expression plasmids pYS-PA-U2-K397D, pYS-PA-U2-K397Q, and pYS-PA-L1-D426K, were transformed into the *E. coli* SCS110 strain, which is dam^-^ and dcm^-^. The purified, non-methylated plasmids from SCS110 were then transformed into the electrocompetent *B. anthracis* BH480 strain, which was plated on LB-agar containing 20 μg/mL kanamycin. BH480 is an avirulent large plasmids-cured, sporulation-defective *B. anthracis* strain [23, 24], serving as an efficient host for recombinant protein expression. Single colonies were grown overnight in 5 mL FA medium containing 20 μg/mL kanamycin. The supernatants containing the mutant PA proteins were sterilized by centrifugation, concentrated ∼10 fold using Amicon Ultra-4 (30 K) Centrifugal Filter Devices (Millipore Corp., Billerica, MA), and were screened for activity in a cytotoxicity assay before purification. PA-L1-D426K and PA-U2-K397Q were further purified for more detailed analyses, as described previously [32].

### Cytotoxicity assay

RAW264.7 murine macrophages, murine B16-BL6 melanoma cells, and murine Lewis lung carcinoma (LLC) cells were grown in Dulbecco’s Modified Eagle Medium (Life Technologies, Grand Island, NY) supplemented with 10% fetal bovine serum (Invitrogen) and gentamycin at 50 μg/mL (Invitrogen) at 37°C in a tissue culture incubator with 5% CO_2_. In cytotoxicity assays, B16-BL6 cells and LLC cells, cultured in 96-well plates at less than 50% confluence, were incubated with various concentrations of PA variants and 100 ng/ml FP59 for 48 h. RAW264.7 cells grown to 50-100% confluence in 96-well plates were incubated with various concentrations of PA variants and 500 ng/ml LF for 5 h. The cells were then incubated with 500 μg/mL MTT (3-(4,5-dimethyl-2-thiazolyl)-2,5-diphenyl-2H-tetrazolium bromide, Sigma, St. Louis, MO) for 30 min. The medium was aspirated and the oxidized MTT was solubilized in 91% isopropanol containing 0.5% SDS and 0.038 M HCl. Absorbance was read at 570 nm using a SpectraMax 190 plate reader (Molecular Devices, Sunnyvale, CA) to evaluate cell viability [35].

### *In vivo* complementing activity of the PA variants

C57BL/6J female mice (10 to 12-week-old) (5 mice per group) were injected intraperitoneally with two doses of various ratios of 20 μg total of PA-U2-K397Q and PA-L1-D426K plus 10 μg FP59 with a two-day interval, and monitored for malaise and survival for two weeks. All animal studies were carried out in accordance with protocols approved by the National Institute of Allergy and Infectious Diseases Animal Care and Use Committee.

### *In vivo* anti-tumor studies

Twelve-week-old female C57BL/6J mice were injected with 5×10^5^ LLC cells in the mid-scapular subcutis. Eight days after injection, established tumors were measured with digital calipers (FV Fowler Company, Inc., Newton, MA). Tumor volumes were estimated using the formula: tumor volume (mm^3^) = ½(length in mm × width in mm × height in mm). Tumor-bearing mice were randomized into different treatment groups and injected intraperitoneally as indicated in Figure 3. Mice were weighed and tumors were measured before each injection. Pentostatin (SML0508) was from Sigma. Cyclophosphamide (NDC10019-957-01) was from Baxter Healthcare (Deerfield, IL).

### Measurement of anti-toxin neutralizing antibodies

LLC tumor-bearing mice from various treatment groups were terminally bled and sera prepared. To titrate anti-toxin neutralizing antibodies in sera, RAW264.7 cells grown in 96-well plates were incubated with 100 ng/mL PA plus 100 ng/mL LF (concentrations that kill >95% of the cells) in the presence of various dilutions of the sera for 5 h, followed by the MTT assay to determine cell viabilities, as described above.
